# Development and characterization of a cancer cachexia model employing a rare human duodenal neuroendocrine carcinoma-originating cell line

**DOI:** 10.18632/oncotarget.26764

**Published:** 2019-03-29

**Authors:** Kazuyoshi Yanagihara, Takanori Kubo, Yuki Iino, Keichiro Mihara, Chie Morimoto, Toshio Seyama, Takeshi Kuwata, Atsushi Ochiai, Hiroshi Yokozaki

**Affiliations:** ^1^ Division of Biomarker Discovery, Exploratory Oncology and Clinical Trial Center, National Cancer Center, Chiba, Japan; ^2^ Department of Life Sciences, Yasuda Women’s University Faculty of Pharmacy, Hiroshima, Japan; ^3^ Department of Hematology/Oncology, Research Institute for Radiation Biology and Medicine, Hiroshima University, Hiroshima, Japan; ^4^ Department of Living Science Nutrition Course, Matsuyama Shinonome Junior College, Matsuyama, Japan; ^5^ Department of Pathology and Clinical Laboratories, National Cancer Center Hospital East, Chiba, Japan; ^6^ Division of Pathology, Department of Pathology, Kobe University Graduate School of Medicine, Kobe, Japan

**Keywords:** angiogenesis, cancer cachexia, duodenal neuroendocrine carcinoma, IL-8, orthotopic animal model

## Abstract

Cancer cachexia interferes with therapy and worsens patients’ quality of life. Therefore, for a better understanding of cachexia, we aimed to establish a reliable cell line to develop a cachexia model. We recently established and characterized the TCC-NECT-2 cell line, derived from a Japanese patient with poorly differentiated neuroendocrine carcinoma of the duodenum (D-NEC). Subcutaneous xenograft of TCC-NECT-2 cells in mice resulted in tumor formation, angiogenesis, and 20% incidence of body weight (BW)-loss. Subsequently, we isolated a potent cachexia-inducing subline using stepwise selection and designated as AkuNEC. Orthotopic and s.c. implantation of AkuNEC cells into mice led to diminished BW, anorexia, skeletal muscle atrophy, adipose tissue loss, and decreased locomotor activity at 100% incidence. Additionally, orthotopic implantation of AkuNEC cells resulted in metastasis and angiogenesis. Serum IL-8 overproduction was observed, and levels were positively correlated with BW-loss and reduced adipose tissue and muscle volumes in tumor-bearing mice. However, shRNA knockdown of the IL-8 gene did not suppress tumor growth and cachexia in the AkuNEC model, indicating that IL-8 is not directly involved in cachexia induction. In conclusion, AkuNEC cells may serve as a useful model to study cachexia and D-NEC.

## INTRODUCTION

Cancer cachexia is characterized by anorexia as well as decreased body weight (BW), skeletal muscle loss, and adipose tissue atrophy. These attributes make therapeutic interventions less effective, and cachexia is responsible for deaths in cancer patients [1, 2]. In cachexia, BW-loss is observed in 30–80% of cancer patients. However, in 15% of the patients, the condition is very severe depending on the tumor type. Specifically, gastric or pancreatic cancer patients show BW-loss at very high frequencies, whereas BW-loss is less prominent in patients with breast cancer, leukemia, or sarcomas [3, 4].

The cachexia-causing mechanism is considered the result of a complex interplay between tumor and host factors [5]. Cachectic patients with gastroenteropancreatic carcinomas (GEPCs) and tumor-bearing experimental animals exhibited increased plasma cytokines, such as interleukin-1β (IL-1β), interleukin-6 (IL-6), interleukin-8 (IL-8), interleukin-10 (IL-10), leukemia inhibitory factor (LIF), TNF-α, and VEGF-A, which are either produced by cancer cells or released by the host immune system in response to cancer [4, 6–8]. In addition, circulating ghrelin levels have been reported to increase in cachectic patients with gastric and neuroendocrine tumors (NETs)[9, 10]. Although ostensibly induced by multiple tumor-produced cytokines, their functional contribution to the development and/or progression of cachexia has not been fully elucidated.

Although research on the pathophysiology of cancer cachexia is accelerating, there is a need for additional and better-quality studies. Experimental animal models have provided insight into mechanisms, but the translational value of cancer cachexia animal models requires confirmation. A large proportion of prior research, such as those on colon 26 adenocarcinoma, Lewis lung carcinoma, Yoshida hepatoma, and Walker 256 carcinosarcoma, has been conducted on a rather limited population [11]. For these tumors, the original cell lines have become unavailable; however, cells have been distributed among different labs. Currently available subclones are likely to have transformed over time, thus they do not provide consistent results [12, 13]. Therefore, generation of new cachexia models, aligned with the clinical cachexia isotypes, will offer new avenues for pre-clinical investigations. For example, a murine model of pancreatic cancer, KRAS^G12D^/+ P53-/- Pdx-Cre (KPC) congenic allografts in C57BL/6 mice, recapitulates cachexia features specifically associated with pancreatic ductal adenocarcinoma [14, 15]. Further, development of human cachexia cancer animal models derived from a specific organ is extremely important to clinically align the cachexia model in a specific cancer type. Xenograft mouse models of an established GEPC cell line, which show human-like tumor progression and cachectic behavior, have helped to identify the disease process and develop novel therapeutic approaches [16, 17]. Previously, our reports of orthotopic implantation (OI) of gastric carcinoma cells have shown that subsequent tumor growth results in metastases to various organs and peritoneal dissemination, similar to human cases [18]. We have recently reported on a model of cancer cachexia using OI from human gastric carcinoma cells. This model effectively mimicked the tumor/host interaction and pathogenesis [19].

One of the aggressive GEPCs, an extremely rare neuroendocrine carcinoma of the duodenum (D-NEC), is a rapidly progressing disease that frequently metastasizes to regional lymph nodes and the liver, and is associated with a very poor prognosis [20–22]. The lack of adequate experimental models for human D-NEC [23] prompted us to seek new cell lines that could be used to study pathogenesis and to develop a cachectic model of this tumor. More recently, we established and characterized a cell line TCC-NECT-2, derived from a Japanese patient with poorly differentiated D-NEC [24]. Subcutaneous (s.c.) xenograft of TCC-NECT-2 cells into mice resulted in tumor formation and BW-loss at an incidence of 20%. Moreover, in this article, we report the isolation of a cachexia-inducing subline from the TCC-NECT-2 parent cell line and development and characterization of a cachexia mouse model.

## RESULTS

### Isolation and characterization of cachexia-inducing subline, AkuNEC

**Figure 1:**
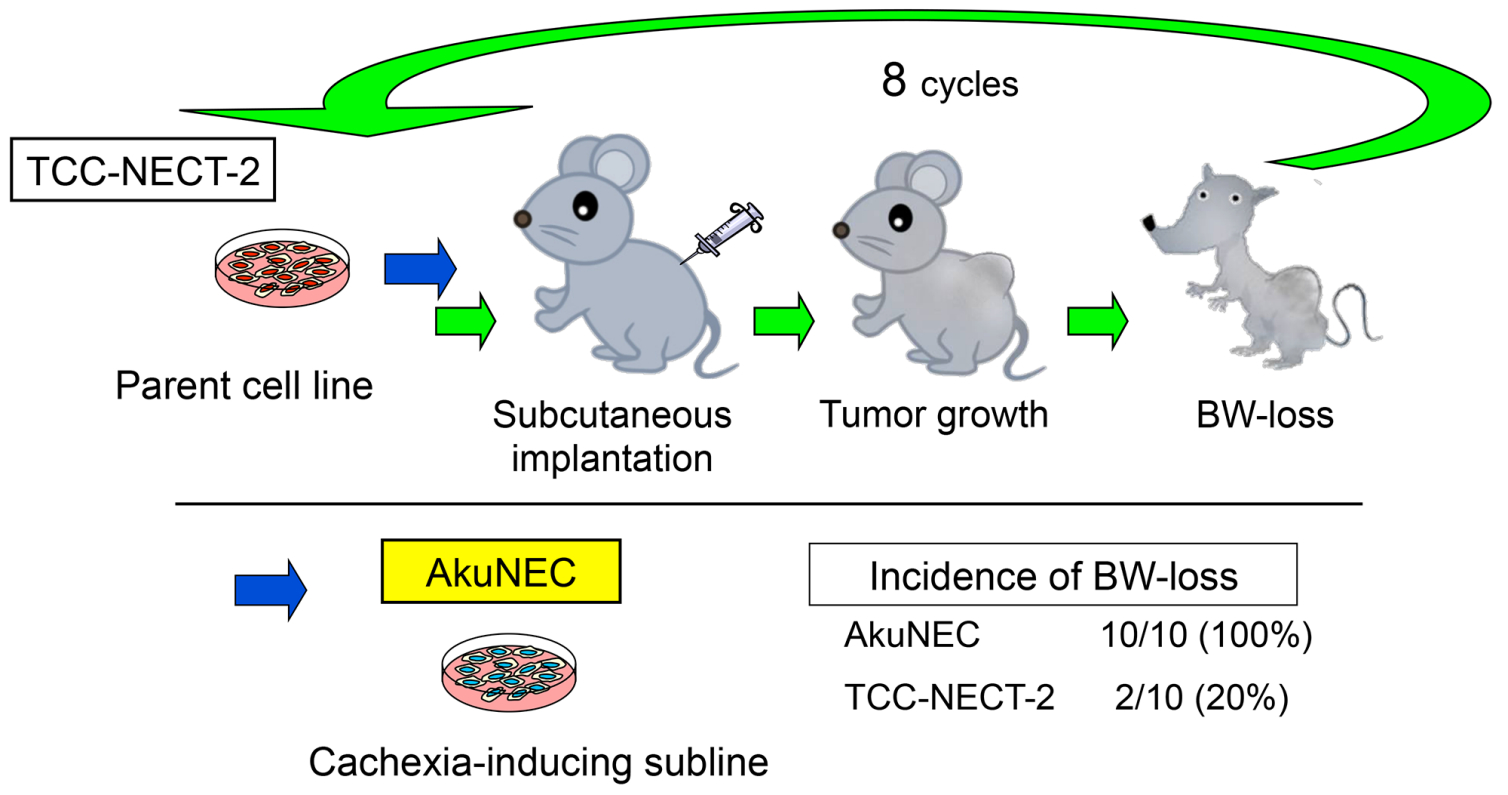
Isolation of subline with potential to cause BW-loss in tumor-bearing nu/nu mice Schematic of cachexia-inducing subline isolation. A highly incident BW-loss induced tumor subline (AkuNEC) with a strong capability of inducing cachexia was isolated through stepwise selection. Specifically, cachexia-inducing TCC-NECT-2 cells were repeatedly injected via s.c. implantation in nu/nu mice. BW-loss is a parameter of cachexia, and details are described in the Materials and Methods section.

The cachexia-inducing subline was isolated according to the scheme shown in Figure 1. Following s.c. inoculation, only 20% of the TCC-NECT-2 tumor-bearing mice showed BW-loss as described previously [24]. Therefore, a highly competent cachexia-inducing tumor cell line was isolated from the TCC-NECT-2 human D-NEC cell line according to the protocol described in the Materials and Methods section. Cycles of isolation of the BW-loss induced tumor cells and s.c. implantation of TCC-NECT-2 cells were repeated in nu/nu mice. This allowed isolation of a highly incident BW-loss induced tumor subline with a strong capability of inducing cachexia, which was designated as AkuNEC.

**Figure 2:**
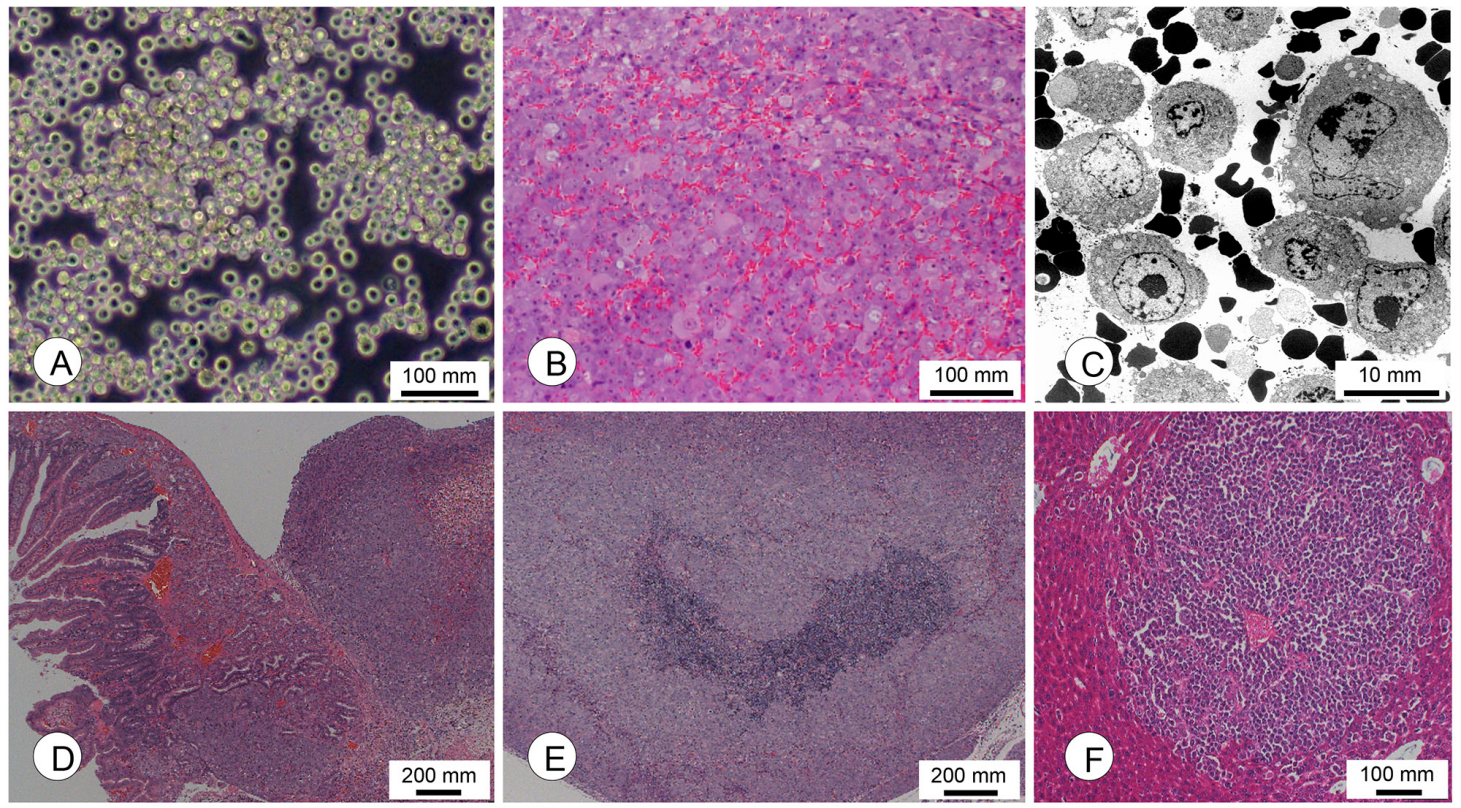
Morphological characterization of cachexia-inducing AkuNEC cell lines **(A)** Phase-contrast photomicrographs of AkuNEC cells. Scale bar: 100 μm. **(B)** Photomicrographs of s.c. tumors in the recipient nu/nu mice following s.c. injection of AkuNEC cells. A wide range of angiogenesis in s.c. tumors was observed. Scale bar: 100 μm, HE staining. **(C)** Electron microscopic observation of AkuNEC s.c. tumors revealed single tumor cells and many erythrocytes. Scale bar: 10 μm. **(D)** Micrographs of the intra-duodenal tumor at 50 days post-OI. Scale bar: 200 μm, HE staining. **(E)** Lymph node metastasis. Scale bar: 100 μm. HE staining. **(F)** Micrometastasis in the liver at 50 days post-OI. Scale bar: 100 μm, HE staining.

AkuNEC cells were round and floating freely, and they tended to form loose, non-adherent multicellular aggregates (Figure 2A). The doubling time was approximately 31.0 hours in RPMI1640 medium supplemented with 10% FBS. This cell line was strictly anchorage-independent (88.5% efficiency) and was able to grow in chemical-defined medium (CDM) (Table 1) [19]. The Ki-67 index was 75.9% (Table 1).

**Table 1:** Biological characterization of the newly isolated AkuNEC cell line from TCC-NECT-2 duodenal neuroendocrine carcinoma parental cell line

Cell line	Growth^a^			Neuroendocrine tumor marker^b^						Cytokine^c^		
	DT (h)	In CDM/agar	Ki-67 index (%)	NSE (ng/mL)	CGA	CD56/NCAM	SYN/p38	SSTR	RB	IL-6 (pg/mL)	IL-8 (pg/mL)	VEGF (pg/mL)
AkuNEC	31.0	(+ /88.5)	75.9	10.5	(+)	(+)	(+)	(-)	(-)	2.3	11,500	4,150
TCC-NECT-2	31.4	(- /63.2)	68.4	5.2	(+)	(+)	(+)	(-)	(-)	ud	7,510	ud

^a^: DT, the doubling time of each line was determined. Chemical-defined medium (CDM), composed of DMEM/Ham’s F-12 (1:1) medium supplemented with 0.05% BSA; (+), positive; (-), negative; ud, undetectable. Colony formation on semisolid agar was assayed by plating 10^3^–10^4^ cells in DMEM containing 10% FBS and 0.33% Difco noble agar. The number of colonies formed was counted 21 days after cell plating (PE, %). Ki-67 expression was detected by IHC staining as described in the Materials and Methods section.

^b^: Secretion of NSE was tested in culture fluids by CLEIA as described in the Materials and Methods section. Expression of CGA, CD56 (NCAM), SYN (p38), SSTR, and Rb was detected by IHC staining.

^c^: Secretion of IL-6, IL-8, and VEGF was tested in culture fluids by CLEIA at SRL Laboratories (Tokyo, Japan). The supernatant was collected from 48-h cultures.

AkuNEC cells exhibited strong positivity for neuroendocrine tumor markers, such as neuron specific enolase (NSE), chromogranin A (CGA), cluster of differentiation 56 (neural cell adhesion molecule; CD56/NCAM), and synaptophysin (major synaptic vesicle protein p38; SYN/p38), but not somatostatin receptor (SSTR) and retinoblastoma (RB) protein by immunohistochemical (IHC) staining analysis, similar to the parental cell line (Table 1). AkuNEC cells secreted tumor marker CA19-9 (21.3 ± 6.6 units/mL), but CEA and CA125 antigen production was not detected (data not shown).

AkuNEC cells secreted IL-8, VEGF, and small amounts of IL-4 (3.5 pg/mL) or IL-6 (2.3 pg/mL) in the culture supernatants. In particular, AkuNEC cells secreted large amounts of IL-8, while IL-8 was secreted to a smaller extent and VEGF was undetectable in TCC-NECT-2 parental cells. These biological characteristics are summarized in Table 1. Production of the following cytokines was not observed in these cells: IL-1β, IL-2, IL-3, IL-10, VEGF, hepatocyte growth factor (HGF), and TP53 (data not shown).

We confirmed the identity of the established cell lines by short tandem repeat (STR) genotyping analysis comparing specific regions of the DNA from the AkuNEC subline and TCC-NECT-2 parent cell line. All DNA extracted from the two established cell lines showed identical STRs, which did not correspond to cells in the Japanese Collection of Research Bioresources database (JCRB; a database of 2279 cells registered in the ATCC, the Deutsche Sammlung von Mikroorganismen und Zellkulturen, and the Japanese Collection of Research Bioresources).

### Molecular biological characterization of AkuNEC and TCC-NECT-2 cell lines

We performed next-generation sequencing (NGS) analyses using the NCC oncopanel (v4) of 114 genes, including oncogenes and tumor suppressor genes. Mutations in BRAF^V600E^ and TP53 (Splicing783-1G>A) genes, as well as amplification of C-MYC (56.9-fold), were detected in AkuNEC cells. Further, the TP53 gene harbored splicing mutations in both alleles. No genetic alterations specific to AkuNEC cells was observed, and it was almost similar to the parental TCC-NECT-2 cell line [24].

### Tumorigenicity, metastatic potential, and BW-loss following different implantation routes of AkuNEC cell line

Tumor formation was noted after injection of AkuNEC cells via s.c., i.p., duodenal, and rectal routes to the mice at incidences of 100%, 87.5%, 100%, and 75%, respectively. The mean survival period was 37.7 days for s.c., 38.8 days for i.p., 40.8 days for OI, and 42.5 days for rectal implantation.

The histological growth pattern of xenografts was a poorly differentiated neuroendocrine carcinoma (polymorphic medullary type) (Figure 2B and Table 2). Extensive angiogenesis was observed in the xenografts (Figure 2B). AkuNEC s.c. tumors were predominantly single cells or free tumor cells and many red blood cells at the ultrastructural level, which are characteristics of typical angiogenesis as shown in Figure 2C.

**Table 2:** Tumorigenicity, metastasis, and BW-loss following different implantation routes of AkuNEC cell line

Cell line	Implanta-tion routes	Tumor formation^a^			Cachectic BW-loss (%)	Metastasis and Invasion				
		Frequency (%)	Survival day	Histological pattern of xenografts		Peritoneal^b^ dissemination	Pan-creas^c^	Lymph-nodes	Liver^d^	Stomach^e^
AkuNEC	s.c.	10/10 (100)	37.7±5.5	PD- NEC^f^	10/10 (100)	0/10	0/10	0/10	0/10	0/10
	i.p.	7/8 (87.5)	38.8±6.5	PD-NEC	7/7 (100)	1/7	0/7	2/7	0/7	0/7
	Duodenum	6/6 (100)	40.8±10.8	PD-NEC	6/6 (100)	1/6	5/6	2/6	2/6	6/6
	Rectum	6/8 (75)	42.5±6.7	PD-NEC	6/6 (100)	1/6	0/6	1/6	0/6	0/6

^a^: tumorigenicity and metastasis of cell lines were tested by s.c. implantation of 1 × 10^6^ cultured cells suspended in 100 μL of PBS into mice. For i.p. inoculation of tumor cells, 1 × 10^6^ cells in 500 μL of PBS were injected into the mouse abdominal cavity. For intra-duodenum or intra-rectum implantation of tumor cells, aliquots of 1 × 10^6^ cells in 50 μL of PBS were injected into the target organ. The mice were sacrificed on day 55 after tumor cell implantation or when they became moribund, and BW-loss was evaluated. Incidence is reported as fractions; numerators of each fraction indicate positive numbers in the samples (denominators).

^b^: Peritoneal dissemination: peritoneum, mesenterium, diaphragm.

^c^: Micrometastases, including invasion through fusion with duodenal tumor.

^d^: Micrometastasis.

^e^: Invasion from duodenal tumor.

^f^: PD-NEC: poorly differentiated neuroendocrine carcinoma.

Following OI, metastasis to the pancreas, lymph nodes, and liver, and invasion to the stomach were noted at incidences of 16.6–100%. Intra-duodenal tumor mass, its histology, and lymph node metastasis were shown in Figure 2D and 2E. Liver metastasis was only detected in the OI route (Figure 2F). Peritoneal dissemination was observed after i.p. injection in the duodenum and rectum, at incidences of 14.3–16.6%.

In addition, BW-loss was observed in mice bearing AkuNEC xenograft, at a 100% incidence (Table 2). As a result, the cachectic phenotype, such as decreased motility, reduced adipose tissue and musculature volumes, and decreased mass of other organs, including the spleen and liver, was observed in BW-loss mice.

Thus, AkuNEC tumor cells exhibited growth patterns and expression of neuroendocrine tumor markers, and their histopathological and molecular characteristics resembled those of the parental TCC-NECT-2 cells as shown in Table 1 and Figure 2A. A clear difference was observed with regard to cachexia induction, metastases (Table 2), and angiogenesis (Figure 2B).

### Induction of cachexia by heterotopic-implantation of AkuNEC cell line

**Figure 3:**
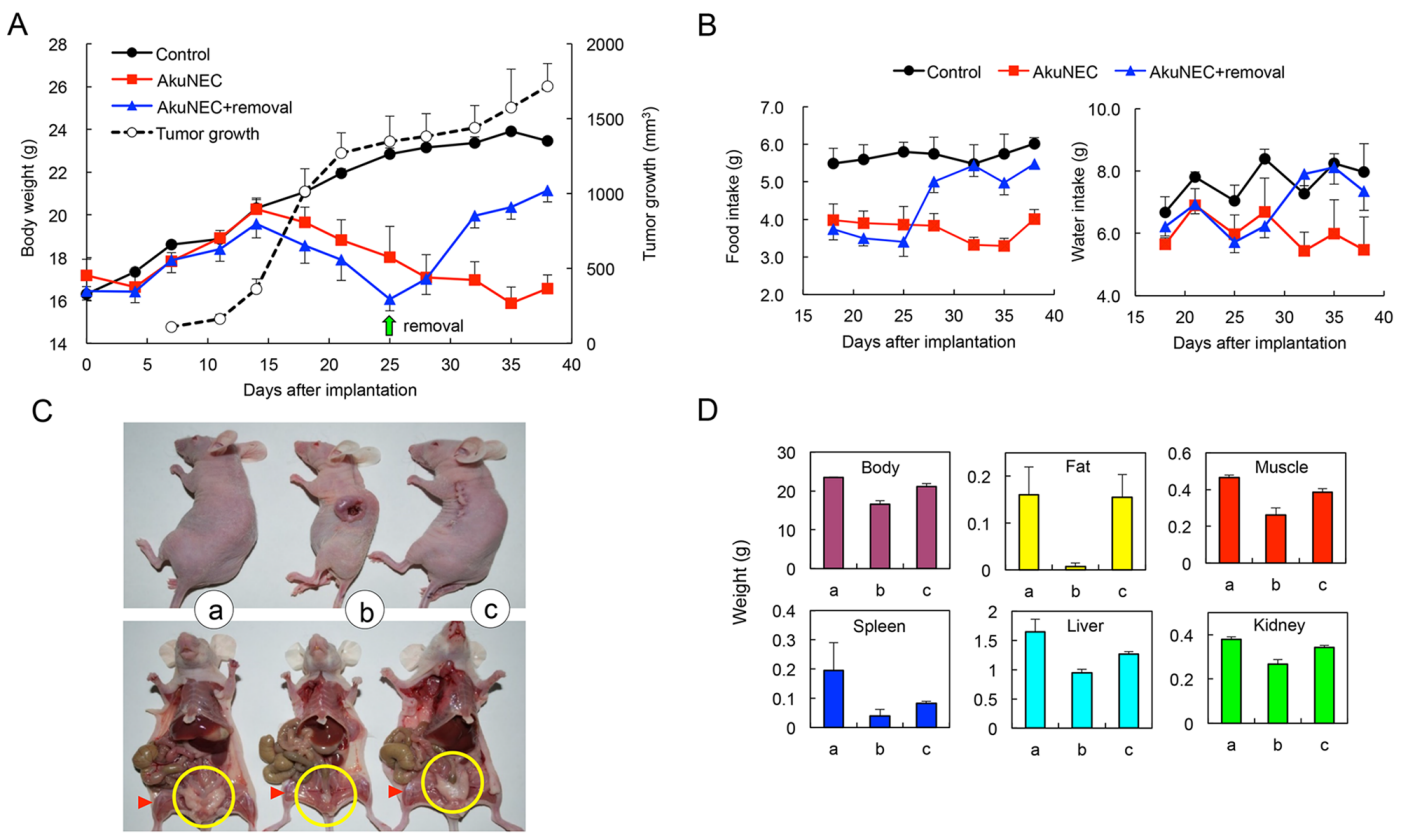
Subcutaneous-implanted AkuNEC tumors induce cancer cachexia **(A)** Growth curves of mouse BW and tumor volume. AkuNEC cells were s.c. implanted into the right flank (2 × 10^6^ cells/each site) on day 0. Growth curve of tumor volume (○). One group of mice carried the tumor during the experiment (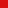). In another group (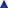), tumors that grew as large as 10 mm in diameter were removed on day 25 (green arrow). The control group consisted of age- and gender-matched mice (●). n = 5. **(B)** Food and water consumption were measured every 3 or 4 days until day 38 from 18 days after implantation of AkuNEC cell line. **(C)** Macroscopic views of mouse autopsies at 38 days after AkuNEC cell s.c. implantation. Both the upper and lower panels consist of age- and gender-matched controls, tumor-bearing, and tumor-removed mice. Apparent changes in the volume of the parametrical fat mass (yellow circle) and hind limb musculature (red triangle) were recognized. n = 5. **(D)** Body weight and each organ weight in histograms of mouse autopsies at 38 days after AkuNEC cell s.c. implantation. In panels, (a) age- and gender-matched control, (b) tumor-bearing, and (c) tumor-removed mice.

BW and food and water intake were examined in mice until 38 days after implantation with AkuNEC cells. Subcutaneous injection of AkuNEC cells induced rapid tumor growth in mice until 20 days after implantation, and then it gradually increased (Figure 3A). The BW of non-tumor-bearing age- and gender-matched control mice continued to increase during the experiment, whereas the BW of mice in the AkuNEC group did not. This BW of AkuNEC-tumor-bearing mice was significantly lower than that in the control group. Food and water intake was examined in mice from 18 days to 38 days after implantation with AkuNEC cells (Figure 3B). Reduced food intake was observed in the AkuNEC group as compared to the control group. A decrease in food intake of the AkuNEC group as compared to control mice was already observed at the initiation of observation at 18 days after implantation, and the difference became slightly greater 38 days later. Water intake was reduced in the AkuNEC group as compared to that in the age- and gender-matched control group. The reduction was stable and persisted to the end of the experiment (Figure 3B). The AkuNEC-tumor-bearing mice appeared to develop a cachectic condition, accompanied by decreased locomotor activity, xeroderma, and anorexia in 100% of mice within 3–5 weeks post-implantation. Further, these mice showed reduced adipose tissue and musculature volumes, as well as decreases in the masses of other organs, including the spleen and liver (Figure 3C and 3D). Moreover, AkuNEC tumor tissues as large as 10 mm in diameter resulted in critical conditions of cachexia in mice on day 25, and surgical removal of the tumors restored health (Figure 3). Thereafter, reduced body weight and food and water intake increased from 3 days after the tumor removal and gradually recovered to the control group level (Figure 3A and 3B). Moreover, reduced adipose- and muscle-tissues, as well as decreases in the masses of other organs, were restored to the normal control state (Figure 3C and 3D).

### Induction of cachexia by OI in AkuNEC cell line

**Figure 4:**
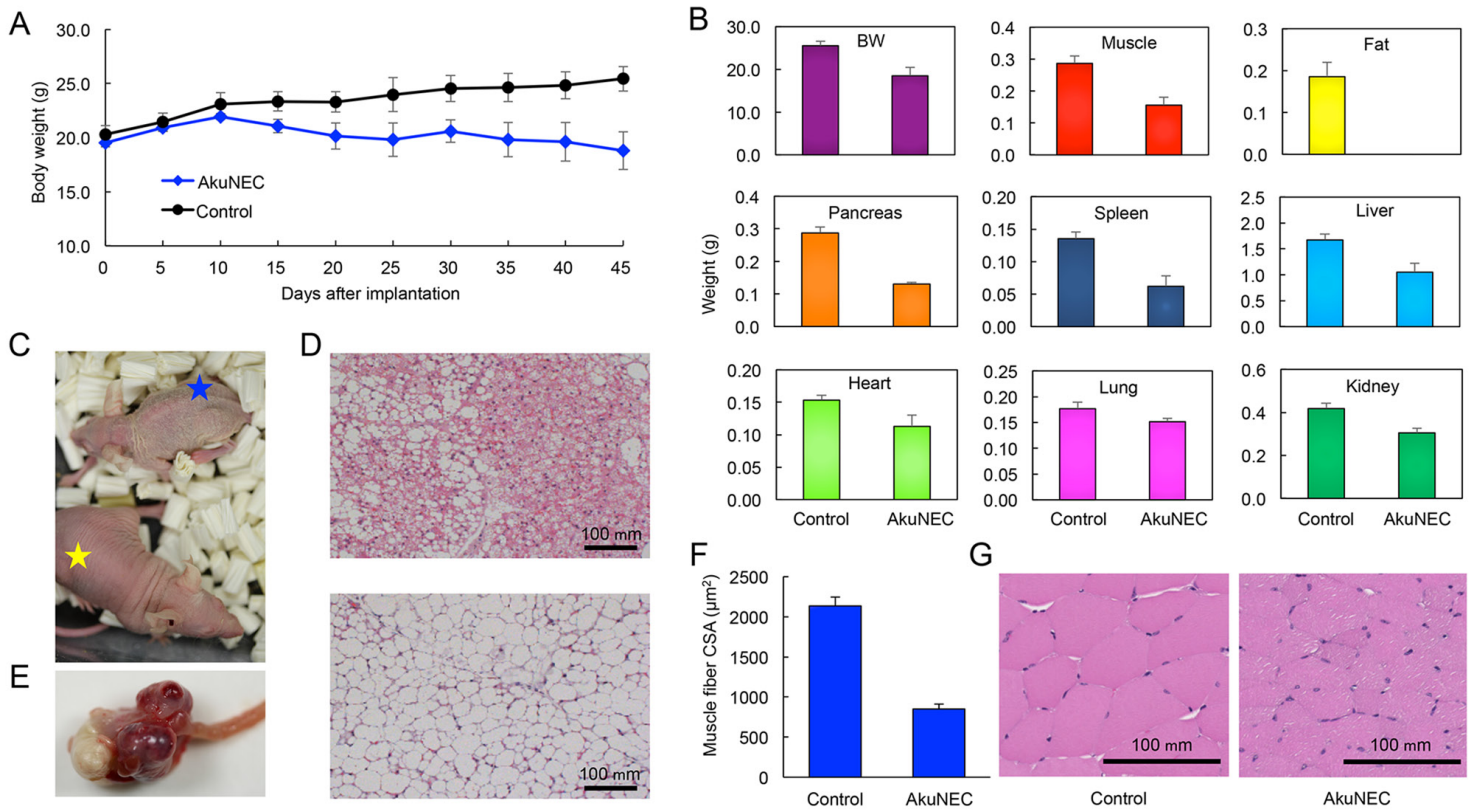
Orthotopically implanted AkuNEC tumors induce cancer cachexia **(A)** Growth curves of mouse BW. AkuNEC cells were implanted into the duodenum (1 × 10^6^ cells) on day 0. One group of mice carried the tumor during the experiment (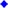). The control group consisted of age- and gender-matched mice (●). n = 5. **(B)** Body weight and each organ weight in histograms of mouse autopsies at 45 days after OI of AkuNEC cells. In panels, (left) age- and gender-matched control and (right) tumor-bearing mice. **(C)** Macroscopic views of an AkuNEC-tumor-bearing mouse (blue star) and an age- and gender-matched sham control mouse (yellow star) at 45 days after OI of AkuNEC cells. **(D)** Representative HE sections of adipose tissue are displayed for mice bearing AkuNEC tumors (upper) compared to the sham control (lower). **(E)** Macroscopic views of an intra-duodenum AkuNEC tumor at 45 days post-implantation. **(F)** CSA of the thigh muscle fibers of AkuNEC-tumor-bearing mice. The mean CSA on day 45 in AkuNEC- tumor-bearing mice and negative gender- and age-matched control mice (received sham surgery) was 849.9 ± 58.4 μm^2^ and 2135.0 ± 108.2 μm^2^, respectively (p < 0.01). The muscle fiber CSA of 200 muscle fibers per muscle was measured. This is the average value of 200 muscle fibers in 10 fields of six (2 × 3) thigh muscles on both sides of three mice. **(G)** Representative HE sections of thigh muscle are displayed for mice bearing AkuNEC tumors (right) compared to the sham control (left).

For OI of AkuNEC cells, BW and food and water intake were examined in mice until 45 days after implantation. Tumor formation in mice was observed by palpation from 2 weeks after implantation, and the tumors were gradually increased. The BW of non-tumor-bearing control mice continued to increase during the experiment, whereas the BW of mice in the AkuNEC group did not (Figure 4A). BW-loss was gradually observed from around 15 days post-implantation and decreased by 25% on the 45th day compared to the age- and gender-matched control group (Figure 4A and 4B). Food and water intake was examined in mice from 5 days to 45 days after implantation with AkuNEC cells. A decrease in food intake of the AkuNEC group as compared to control mice was observed at 10 days after implantation, and the difference was marginal, but stable until the end of the experiment. Water intake was also reduced in the AkuNEC group as compared to the age- and gender-matched control group. The reduction was slight, but it was stable and persisted to 45 days (data not shown).

The group appeared to develop a cachectic condition, accompanied by decreased locomotor activity, xeroderma, and anorexia in 100% of the mice (Figure 4C). When AkuNEC-tumor-bearing mice were sacrificed and necropsied at 45 days post-implantation, tumor formation was clearly observed in the duodenum of mice (Figure 4E). Moreover, the xenograft of AkuNEC cells in the mice reduced adipose tissue and musculature volumes, as well as showed decreases in the masses of other organs, including the spleen and liver (Figure 4B). Histopathologically, adipocytes were miniaturized, and brown adipocytes appeared in AkuNEC-tumor-bearing mice (Figure 4D, in upper panel). The weight and cross-sectional area (CSA) of the thigh muscle fibers of AkuNEC-tumor-bearing mice were significantly smaller (p < 0.01) than those of control mice, confirming muscle wasting (Figure 4F and 4G).

### IL-8 gene knockdown on tumor growth and BW-loss in s.c. implantation of AkuNEC cell line

**Figure 5:**
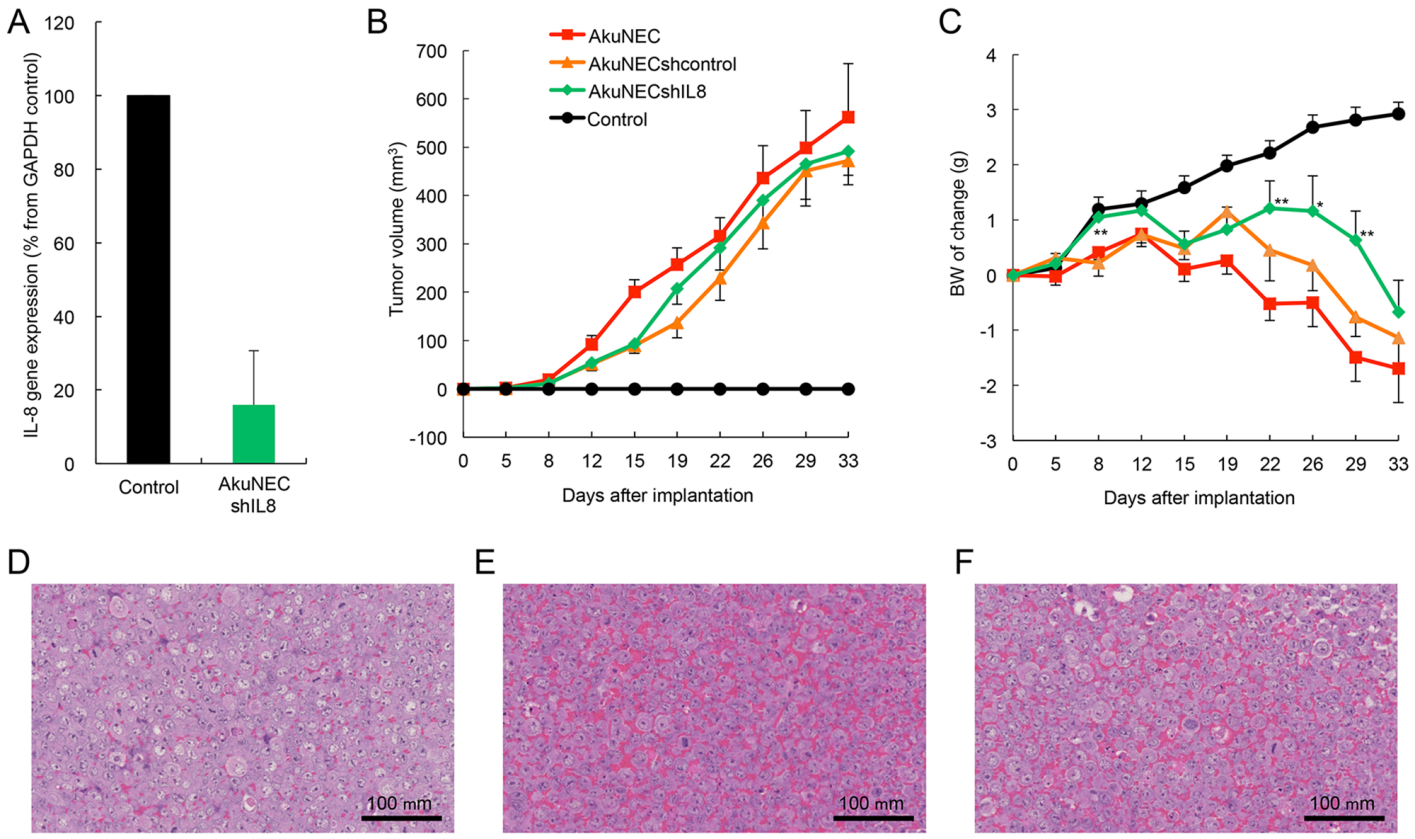
Results of IL-8 gene knockdown on tumor growth and BW-loss in s. c. implantation of AkuNEC cell line **(A)** IL-8 expression was eliminated by gene knockdown in the AkuNEC cell line using vector-based short-hairpin type RNAi. **(B)** Changes in tumor volume and **(C)** body mass in mice that received s.c. implantation of AkuNEC cells (1 × 10^6^ cells/each site) into the right flank on day 0. Tumor growth was evaluated by measuring tumor diameters with a two-dimensional caliper. Tumor volume (TV) was calculated according to the following formula: TV = (L × W^2^ / 2), where V = volume (mm^3^), L = length (mm), and W = width (mm). This was reported as the mean value of eight mice per group. The mean BW was 22.3 ± 1.7 g prior to implantation. In AkuNECshIL8-cells implanted mice, a significant difference was observed on 8, 22, 26, and 29 days after implantation between AkuNEC-implanted groups (^**^p < 0.01 and ^*^p < 0.05, respectively). TV and BW were measured at 0, 5, 8, 12, 15, 19, 22, 26, 29, and 33 days after implantation. AkuNECshcontrol cell- (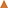), AkuNECshIL8 cell- (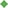), AkuNEC cell-implanted mice (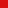), and age- and gender-matched control (●). **(D)** Micrographs of the AkuNECshIL8, **(E)** AkuNECshcontrol,and **(F)** AkuNEC s.c, tumor at 33 days post-implantation. Scale bar: 100 μm, HE staining.

As mentioned previously, high levels of IL-8 production were observed in the sera of AkuNEC-tumor-bearing mice. Moreover, we found that the IL-8 level was positively correlated with the cachectic phenotype, such as BW-loss, anorexia, muscle atrophy, reduced adipose tissue, and decreased locomotor activity (Table 3). Therefore, IL-8 expression was eliminated by knockdown in the AkuNEC cell line using vector-based short-hairpin type RNA interference (RNAi) (Supplementary Table 1). A highly competent IL-8-knockdown stable cell line was isolated using the protocol described in the Materials and Methods section and designated as AkuNECshIL8 (Figure 5A, Supplementary Table 1, and Supplementary Figure 1).

**Table 3:** Correlation between human IL-8 production, BW-loss, anorexia, muscle atrophy, reduced adipose tissue, and locomotor activity of mice

Experiment group	Post-implantation (days)^a^	IL-8 (pg/mL)^b^	Body weight(% of BW-loss)^c^	Anorexia^d^	Muscle atrophy^e^	Reduced adipose tissue^e^	Reduced locomotor activity^f^
Control	38	ud	23.50±0.11 (0.00)	0/4	0/4	0/4	0/4
Tumor-bearing	14	0.7–2.1	21.87±1.48 (6.94)	2/4	1/4	2/4	1/4
	20	5.0–16.1	20.43±1.61 (13.06)	3/4	2/4	3/4	2/4
	36	>25.0	18.63±2.54 (20.72)	4/4	4/4	4/4	4/4
Tumor-removed	38	ud	22.23±1.07 (5.4)	0/4	0/4	0/4	0/4

^a^: Correlation between human IL-8 production and cachexia-associated phenotypes of cell lines was tested by s.c. implantation of 2 × 10^6^ AkuNEC cells suspended in 100 μL of PBS into mice on day 0. On the scheduled day, mice were sacrificed, and sera were collected. Further, BW-loss, muscle atrophy, adipose tissue reductions, and anorexia were evaluated. Incidence is reported as fractions; numerators of each fraction indicate positive numbers in the tested mice (denominators). n = 4.

^b^: ud, undetectable, below the minimum detection limit of the assay kit. Detection limit: 0.39–25.0 pg/mL. n = 4.

^c^: Percentages of BW-loss of each experimental group are indicated as numerators against denominators of the weight of age- and gender-matched control mice.

^d^: Anorexia: measured food- and water-intake were compared and evaluated against the control group as described in the Materials and Methods section.

^e^: Muscle atrophy and ^e^reduced adipose tissues were macroscopically observed at the time of dissection of the mice, and the organ weight was compared and evaluated against the control group.

^f^: Reduced locomotor activity of tumor-bearing mice was compared and evaluated against the control group as described in the Materials and Methods section.

Subsequently, we examined the effects of IL-8-knockdown on tumor growth and BW in the s.c. implantation of AkuNEC sublines, such as AkuNECshIL8, AkuNECshcontrol, and AkuNEC positive control cells. Implantation of AkuNECshIL8-cells proliferated similar to AkuNECshcontrol and AkuNEC cell lines, leading to tumor formation, with no significant change observed among the three experimental groups (Figure 5B). Moreover, the Ki-67 index of the AkuNECshIL8-tumor and others was observed on day 33 by IHC: AkuNECshIL8-tumor, 66.96 ± 4.95%; AkuNECshcontrol-tumor, 67.73 ± 6.06%; and AkuNEC tumor, 68.34 ± 5.55% (n = 8).

Furthermore, AkuNECshIL8-cells implanted mice showed a tendency to increase BW compared to AkuNEC-implanted mice. A significant difference was observed on 8, 22, 26, and 29 days after implantation between AkuNEC-implanted groups (Figure 5C). However, BW decreased similarly to AkuNECshcontrol- and AkuNEC-implanted mice. Low human IL-8 levels were detected in the sera of AkuNECshIL8-tumor-bearing mice (0.3–1.1 pg/mL) compared to AkuNECshcontrol-tumor and AkuNEC- tumor-bearing mice (>25 pg/mL, respectively) on day 33. With regard to mice implanted with AkuNECshIL8-cells, cachexia and associated skin dryness, decreased activity, and anorexia were observed. The mean BW on day 33 in the AkuNECshIL8-tumor-bearing mice, AkuNECshcontrol- and AkuNEC-implanted mice, and negative gender- and age-matched control mice (received sham surgery) were 23.6 ± 2.07, 21.1 ± 1.72, 20.6 ± 1.31, and 25.8 ± 2.11 g, respectively (Figure 5C, p < 0.01).

In histological examination, angiogenesis was clearly suppressed in AkuNECshIL8-tumor-bearing mice compared to AkuNECshcontrol- and AkuNEC-implanted mice (Figure 5D, 5E and 5F). In addition, micrometastasis of the liver was not detected in AkuNECshIL8-tumor-bearing OI mice.

## DISCUSSION

Our study had three major findings. First, we isolated a cachexia-induced subline AkuNEC from a human NEC cell line of duodenal cancer and established a cachexia mouse model. Second, we characterized a cachexia-induced AkuNEC cell line and its heterotopic- and orthotopic-implantation mouse model. Third, we observed the phenomenon that cachexia progression correlated with IL-8 production levels, and we performed an IL-8 gene knockdown experiment and confirmed that IL-8 expression does not directly participate in cachexia induction.

To date, many cachexia animal models have been developed, but limits such as inability to ensure reproducibility or not reflecting the pathophysiological condition of humans are also known [11–13]. Depending on the purpose of the research, selection of an appropriate experimental model should be considered. Syngeneic models, such as the recently reported KPC mice, are considered suitable animal models for studies, such as involvement of immunity in cachexia [14, 15]. Further, a xenograft model in which human cancer cells were implanted into immunocompromised animals is a meaningful human cancer-mimicking model [25]. We have already established a cachexia-inducing subline, 85As2, from the MKN-45 cell line and developed a mouse model of gastric cancer with high frequency cachexia [19]. Subsequently, 85As2mLuc cells were implanted into nude rats and acclimatized, and the characteristics of this model, drug sensitivity, and efficacy were reported [26–28]. For pancreatic cancer with high frequency cachexia, relatively large numbers of cachexia models are known [14, 15, 17, 29, 30], but few studies have been conducted on cachexia associated with NEC. To our knowledge, there is only one case report [31].

NEC is a highly malignant rare carcinoma that occurs in various organs, such as the digestive and respiratory organs, and an increasing incidence has been reported in recent years [32]. D-NEC is a very rare cancer (incidence rate 0.06–2.9%) among digestive cancers [33, 34]. In particular, among NEC cases with a primary tumor arising from the digestive tract, case reports of D-NEC that are not papillary carcinoma are infrequent. Further, the genetic changes and carcinogenic mechanism are unknown. Research into new treatment options is ongoing; however, these options still need to be standardized [35–38]. A D-NEC derived cell line would be indispensable for carcinogenic mechanism and molecular target discovery studies; however, this cell line has not yet been described [23]. More recently, we established a TCC-NECT-2 cell line derived from D-NEC [24]. TCC-NECT-2 was derived from an ascites sample of a metastatic duodenum NEC (a poorly differentiated NEC). In clinical information, the patient received short-term chemotherapy (details unavailable) subsequent to the initial diagnosis [39]. Eventually, the patient succumbed to cachexia and died. In this study, a cachexia model AkuNEC was created by xenograft of a unique carcinoma NEC, for which limited cachexia-associated research has been conducted. To date, there is no published literature that has established a cachexia model of human NEC derived from any other organ. Therefore, our AkuNEC cachexia-inducing subline derived from TCC-NECT-2 cells will be a valuable asset as an experimental cachexia model of basic and preclinical research.

First, we conducted sequence analysis on genetic alterations between newly isolated AkuNEC cells and the parental TCC-NECT-2 cell line, which has a low frequency of cachexia induction. Unfortunately, as described in the Results section, although abnormalities such as mutation and gene amplification common to both cells could be detected, no genetic alteration specific to the cachexia-inducing AkuNEC subline was observed. Therefore, we would like to analyze gene and protein expression between these cells in future studies.

Combinations of tumor side factors and the host side response are considered key factors to the pathogenesis of cachexia [40]. Loss of the spleen and liver in tumor-bearing animals was observed in this AkuNEC and the gastric cancer 85As2 models [19], which was commonly observed in human cancer xenograft cachectic models against immunodeficient mice and rats [26]. These findings are in contrast to the data of rodent immunocompetent tumor models and cancer patients [15, 17]. Syngeneic and genetically engineered mouse models are known to provide immunological and microenvironment parameters, and the presence or absence of host immune cells and stromal cells is thought to be largely responsible for the cachexia phenotype. Thus, the cachectic phenotype in the AkuNEC xenograft model appears to be more dependent on the implanted tumor than the host response.

In addition, the phenotype of cachexia models was dependent on the implantation route, with OI inducing more severe cachexia than s.c. implantation [14, 15, 41]. In xenograft models lacking an intact immune system, Delitto et al reported that both flank and orthotopic pancreatic tumors produced a proinflammatory systemic environment contributing to muscle wasting with a more substantial effect in OI mice [14]. This indicates that the proinflammatory systemic environment is established by tumor growth at the primary site, even without the tumor microenvironment of immune and stromal cells, strongly suggesting the importance and significance of OI in xenografts. In contrast to the results of these pancreatic cancer models, in the AkuNEC model, weight loss occurred irrespective of the primary site of tumor growth (orthotopic and ectopic), and survival showed a similar tendency. Further, in this regard, the cachexia phenotype of AkuNEC is thought to be dependent on the transplanted tumor rather than through the host response. Considering the above findings, this suggests the existence of a new mechanism of cachexia development that is highly dependent on tumor side factors and less involvement of the host microenvironment.

The AkuNEC model presented by us should clarify an important point. Tumor burden (5–10% of body weight) against cachexia induction is high, which is a nonphysiological condition and does not occur in humans. Therefore, it is necessary to clarify whether cachexia is caused with a lower tumor burden (< 5%). As described above, the cachexia-inducing human gastric cancer subline 85As2 also had a high tumor burden rate in nude mice [19] and was transplanted into nude rats to establish a proper cachexia model and/or for animal welfare [26]. In this rat model, the tumor burden rate was low, and it was able to withstand drug evaluation as a suitable animal model [27, 28]. As such, a similar approach is considered more effective for AkuNEC cells.

NEC metastasizes to lymph nodes and the liver, and with extreme metastasis, it has an extremely poor prognosis [33, 42]. The high Ki-67 index, proliferation in CDM, and high colony forming ability indicate that the selected AkuNEC cells are a highly malignant subline. When implanted orthotopically to mice, the metastatic and invasive potentials were high. As cells reflect stable biological properties, high reproducibility, and the malignant stage of D-NEC, the AkuNEC model will also attract attention as a useful metastasis model [43]. The stability of the phenotype in the experimental model is extremely important. We performed a comparative analysis with parental cells by NGS on whether the AkuNEC cell phenotypic stability was due to mutation in the stepwise selection process. As a result, point mutations specific to AkuNEC cells were not detected, suggesting that genetic mutation is less likely to be involved in maintaining stability. The mechanism to stabilize the phenotype is a future task. However, careful handling of cells is considered important for maintaining characteristics of the established cells (quality control). Therefore, we assume that cell culture passages can change their characteristics, and the passage is up to 3 months. In addition, freezing a large amount of established cultured cells (at approximately 10th passages) and thawing is necessary.

AkuNEC cells transplanted into the duodenum site showed peritoneal dissemination as well as metastasis to the liver and lymph nodes. However, it is unclear why metastasis is only observed in the OI. The OI model is well established to be more clinically relevant than ectopic inoculation models (such as s.c., i.p., and i.v.) because of the interaction between tumor cells and the specific organ microenvironment, despite species differences. Further, it mimics many important biological features of cancer progression, metastasis, angiogenesis, and drug resistance [16, 44–48]. Differences also exist in tumor biology and morphology in the xenograft site [16, 48, 49]. However, details of the host microenvironment and metastasis due to OI are still unclear. Molecules expressed during specific interactions of cancer cells with interstitial cells in developing organs may promote metastasis, and it is expected that they will become more clearly defined in the rapidly progressing exosome research [50, 51]. Our preliminary ultrastructural observation confirmed the existence of numerous exosome-like small granules outside the tumor cells at the OI site, which will be explored in a future study.

Cancer cachexia is characterized by loss of body weight and muscle, adipose tissue wasting, and systemic inflammation. Even in experimental tumors, a murine model implanted with adenocarcinoma tumors reported changes in adipose tissue, including shrunken adipocytes and decreased expression of adipose tissue transcription factors [52]. Recent studies demonstrated the ability of white adipose tissue (WAT) to be induced toward the brown adipose tissue (BAT) phenotype (beige or brite adipose tissue) in a process referred to as ‘‘browning” [4, 53]. It has been reported that activation of BAT (via increased Ucp1 expression) contributed to the development of cachexia in mice with colon cancer cell line implantations [54]. Petruzzelli et al found that WAT browning occurred in the initial stage of cachexia and contributed to energy consumption and increased lipid recruitment [55]. Increased beige cell formation was observed in several cancer cachectic mouse models, indicating that this is a consistent feature of cachexia [55, 56]. Even in our model, adipose tissue in the cachexia state was atrophied, adipocytes were smaller and changed to brown, and this finding was consistent with the above reports. WAT browning was also observed in adipose tissues of cachectic cancer patients and has been suggested to be important in cancer cachexia in humans [55, 57]. From a clinical perspective, a critical component of body mass loss observed in cancer cachexia is the depletion of muscle mass [58]. Muscle atrophy was also observed in our model, resulting from reduced protein synthesis (anabolism), increased protein degradation (catabolism), or a combination thereof [59].

Inflammation is the driving force of cachexia, and the most commonly implicated proinflammatory cytokines in cachexia include tumor necrosis factor α (TNF-α), interleukin (IL) 1, IL-6, and IL-8 [60]. Although it has been reported that various cytokines are involved in cachexia induction, the mechanism is not fully understood [6–10]. Therefore, we compared humoral factors, such as cytokines found in AkuNEC and parent TCC-NECT-2 cells, and determined that AkuNEC cells produce a large amount of IL-8. The serum levels were positively correlated to cachectic behavior. Knockdown of the IL-8 gene did not affect tumor growth, Ki-67 index, and weight changes in the AkuNEC mouse model. This finding suggests that IL-8 is not directly involved in cachexia induction, instead a new induction mechanism might exist. To date, much effort has been directed to improve understanding of the mechanisms involved in the complex metabolic syndrome of cachexia. Involvement of exosomes, including microRNA (miRNA) and muscle-specific miRNA (myomiR), has been proposed as one of the mechanisms that can participate in the transduction of inflammatory signals and activation of catabolic states in muscle [61]. Cancer-derived exosomes reprogram systemic energy metabolism and accelerate cancer-associated cachexia to promote tumor progression [62]. As described above, a number of exosome-like particles were detected intra-duodenum of the AkuNEC tumor by electron-microscopic observation. Therefore, exosome involvement should be considered in this model.

IL-8 has potent pro-angiogenic properties [63]. By our histopathological observations, the number of intra-tumor blood pools was small in mice transplanted with IL-8-knockdown AkuNEC cells, and this suppression of angiogenesis was thought to inhibit micrometastasis to the liver. These findings suggested that angiogenesis induced by IL-8 is involved in metastasis [64, 65].

In conclusion, we isolated a cachexia-inducing subline AkuNEC from recently established TCC-NECT-2 cells. Furthermore, we developed and characterized a cachexia-inducing mouse model using AkuNEC cells. These *in vitro* and *in vivo* models are promising tools to analyze the pathobiology of the very rare D-NEC, allowing discovery of therapeutic target molecules and the pathogenesis/mechanisms of cancer cachexia.

## MATERIALS AND METHODS

### Ethics statement

Investigation has been conducted in accordance with the ethical standards and according to the Declaration of Helsinki and according to national and international guidelines and has been approved by the Committee for Ethics in Animal Experimentation of the National Cancer Center and Yasuda Women’s University in accordance with Institutional and Japanese Government Guidelines for Animal Experiments.

### Cell line and culture

TCC-NECT-2 cell line was recently established from a poorly differentiated neuroendocrine carcinoma of the duodenum (D-NEC) in our laboratory [24]. All cell lines were maintained in RPMI1640 medium supplemented with 10% heat-inactivated FBS, 100 IU/mL penicillin G sodium, and 100 mg/mL streptomycin sulfate (Gibco, California, USA). They were maintained at 37°C in a humidified incubator under 5% CO_2_. The cell line was routinely tested for Mycoplasma using a PCR Mycoplasma Detection technique at the Central Institute for Experimental Animals (Tokyo, Japan), and no contamination was detected.

### Isolation of cachexia-inducing cell lines

The cachexia-inducing subline was isolated according to the scheme shown in Figure 1. In a preliminary *in vivo* study of tumor growth, TCC-NECT-2 cultured cells (1 × 10^6^ cells in 100 μL of PBS) were inoculated s.c. into nu/nu mice. After confirming tumor growth and BW-loss, the mice were sacrificed, and tumor tissues were removed under sterile conditions for *in vitro* cultivation. The obtained specimens were washed 5 times in RPMI1640 medium containing 500 IU/mL penicillin G sodium and 500 mg/mL streptomycin sulfate. The tumor tissues were trimmed to remove necrotic tissue debris and then minced with an ophthalmic scissors. Subsequently, 10–15 pieces of tissues were explanted into 100-mm culture dishes (Falcon, New York, USA) with 5 mL RPMI1640 medium containing 15% FBS. The dishes were left undisturbed for 10 h at 37°C in a 5% CO_2_/95% air atmosphere. After 10 h, RPMI1640 medium with 10% FBS, 100 IU/mL penicillin G sodium, and 100 mg/mL streptomycin sulfate was added to the dishes. After 7–14 days, floating tumor cells were transferred to new dishes to selectively remove overgrowing fibroblasts. Additionally, half of the volume of culture medium was changed on average every 4th day. Following a 3–4 wk culture, the grown tumor cells (1 × 10^6^) were reimplanted s.c. into mice. Cachexia-inducing tumor cells were removed from these mice, cultured, and then implanted into naive mice. This process was repeated multiple times to isolate a highly potent cachexia-inducing subline, and mice subjected to implantation with selected cells showed cachectic BW-loss. Cells inducing cancer cachexia steadily were isolated after eight cycles of stepwise selection. The newly established subline AkuNEC was used in the present study.

### Tumor markers and cytokines

Tumor cells (1 × 10^6^ cells) were seeded to 100-mm dishes in RPMI1640 medium supplemented with 10% FBS and cultured for 48 h. The medium was then replaced. After 24 h, culture supernatant (1.5 × 10^6^ cells/mL) was collected and centrifuged at 3000 rpm for 10 min to eliminate cell debris. The resultant supernatant was stored at -80°C until use in assays. Concentrations of CA19-9, CA125, CEA, and NSE were determined by the chemiluminescent enzyme immunoassay (CLEIA) technique at SRL Laboratories (Tokyo, Japan). Secretion of IL-1β, IL-2, IL-3, IL-8, IL-10, VEGF, HGF, and TP53 was tested by ELISA at FALCO Biosystems (Tokyo, Japan). Secretion of IL-4 and IL-6 was tested by CLEIA. The results are mean values of triplicate assays (variability less than 10%).

### Short tandem repeat genotyping

STR genotyping was performed using genomic DNA extracted from AkuNEC and TCC-NECT-2 cell lines. This analysis was performed by Promega (Tokyo, Japan). This experiment was conducted using the PowerPlex® 16 System (Promega) according to the manufacturer’s instructions. The cell authentication report number of the cell lines established in this study is KBN 0299.

### Next-generation sequence (NGS)

Genomic DNA extracted from the AkuNEC cell line was prepared with a QIAamp DNA Mini Kit (Qiagen, Hilden, Germany) according to the manufacturer’s protocol. We performed NGS analyses using the NCC oncopanel (v4) for 114 cancer-related genes. Targeted sequencing and data analyses were as previously described [24].

### Animal experimentation

Female BALB/c nu/nu mice were purchased from CLEA Japan (Tokyo, Japan) and maintained under specific pathogen-free conditions. Briefly, 6 to 8-week-old mice were used in this experiment.

For OI, after induction of anesthesia with 5% isoflurane in room air (flow 300 mL/min), mice were maintained in 2% isoflurane anesthesia via a face mask throughout the operation. After sterilization of the abdomen with 70% ethanol and making a small incision in the median abdominal wall under anesthesia, the duodenum was exposed, and 1 × 10^6^ cells in 50 μL of PBS were directly injected into the duodenum using a 30-gauge needle (Nipro Co, Tokyo, Japan). For implantation into the rectum, tumor cells (1 × 10^6^ cells in 50 μL of PBS) were inoculated into the middle wall of the rectum using a 30-gauge needle. The needle was carefully withdrawn to avoid regurgitation along the needle track, and the injection orifice was pressure-sealed with a dry cotton tip. The incised abdominal wall was closed with an AUTOCLIP Applier (Becton Dickinson, Maryland, USA). After confirming recovery from the bradycardia and stable spontaneous respiration, the mice were returned to their cages. The mice were sacrificed at post-surgery 55 days after tumor cell inoculation (endpoint of the experimentation), when tumor volumes reached 2000 mm^3^ (as the limit of the observation period of tumor growth), or when moribund. Further, the abdominal tissues were inspected macroscopically for metastasis in various organs and thereafter processed for histological examination.

### Pathomorphological and IHC analyses

Tumor tissues from mice transplanted with cancer cells were fixed in phosphate-buffered 10% formalin and embedded in paraffin. Sections were cut at 5 μm intervals and stained with Hematoxylin-Eosin (HE) according to a standard histological protocol. Ultrastructural studies were performed on the cells as previously reported [66]. IHC staining was carried out according to the company’s instruction and/or a standard protocol as described previously [24]. The following antibodies were used, including: CGA (1:500) from Neomarker (California, USA); Ki-67 (1:250), serotonin (1:200), cytokeratin (AE1/AE3 1:50), vimentin (M0725 1:100), and synaptophysin (1:200) from Dako (California, USA); somatostatin receptor 2A (1:500) and 5 (1:500) from Gramsch (Schwabhausen, Germany); CD56/ NCAM (1:100) from Novocastra (Newcastle, England); and RB (clone 3H9, 1:300) from MBL (Nagoya, Japan). VECTASTAIN ABC HRP kit from Vector Laboratories (California, USA) was used for analysis. The Ki-67 index was obtained by counting the ratio of Ki-67 positive cells versus total nuclei using VENTANA iScan HT (Arizona, USA).

The thigh muscle fibers were determined as the field area divided by the number of myofibers in HE-stained transverse sections. Images were captured using a NanoZoomer 2.0HT (Hamamatsu Photonics, Shizuoka, Japan), and the muscle fiber cross-sectional area (CSA) of at least 200 muscle fibers per muscle was measured using NanoZoomer Digital Pathology (view 2, U12388-01ver., 2.7/Rev.1) software (Hamamatsu Photonics). Each histological analysis was performed on right and left thigh muscles from 3 mice per group.

### Measurement of BW, food intake, and water intake and locomotor activity

Mice were implanted s.c. with AkuNEC cells in their right flanks (1 × 10^6^ cells/site) on day 0. Tumor tissues grew as large as 10 mm in diameter on day 25. The mice were anesthetized by inhalation of 2% isoflurane, and the tumors were surgically removed. BW, organ tissue weights, and food and water consumption were evaluated in each mouse. BW was reported as total body weight inclusive of the tumor. BW was measured every three days until day 38. Each mouse was housed individually, and food and water consumption were measured every 3 or 4 days until day 38 from day 18 post-implantation of the AkuNEC cell line. The measured quantity was divided by the number of days and plotted as the amount per day.

For OI of AkuNEC cells (1 × 10^6^ cells/site), BW and food and water consumption were measured every five days until day 45 from 5 days post-implantation. Organ tissue weights were evaluated during dissection of each mouse.

For locomotor activity measurement, tumor-bearing and control mice were individually housed in a voluntary wheel running (VWR) cage and were kept for 72 hours from day 3 before scheduled necropsy, and the number of wheel rotations was measured. Tumor-bearing mice with VWR parameters of 50% or less of the control group were considered as reduced locomotor activity. VWR parameters were measured via the wheel running cage (KN-79-A and KN-79-B; Natsume Seisakusho, Tokyo, Japan).

### Isolation of a highly competent IL-8 gene knockdown stable cell line

The lentiviral vector-expressed shRNA for silencing human IL-8 (NM 000584) was designed and synthesized by Sigma-Aldrich. Five individual clones from IL-8 shRNA targets inserted into a lentivirus packaging plasmid (MISSION™ lentiviral transduction particles, SHCLNV, Sigma-Aldrich) were infected in the AkuNEC cell line. Target sequences of the five individual shRNA (shIL8-028, shIL8-029, shIL8-030, shIL8-031, and shIL8-032) for IL-8 are shown in Supplementary Table 1. After 24 hours post-infection, the shRNA lentiviral particle-containing medium was removed and replaced with fresh medium containing puromycin (1 μg/mL) for selection of transduced cells. To analyze shRNA efficacy, cells were harvested, and the mRNA level of IL-8 was detected by real-time PCR (Supplementary Table 2). Glyceraldehyde-3-phosphate dehydrogenase (GAPDH) mRNA was used as an internal control. The AkuNEC cells used in this study that expressed IL-8 targeted shRNA (AkuNECshIL8) were chosen from the clone that showed the highest RNAi potency for IL-8 (shIL8-028). AkuNEC cells expressing non-target shRNA, which were infected by MISSION™ pLKO.1-puro non-target shRNA control transduction particles (SHC016V), were used as control cells (AkuNECshcontrol).

### Statistical analysis

All data were analyzed using the unpaired t-test and expressed as mean ± SEM for *in vivo* analysis. P-values less than 0.05 were considered statistically significant.

## SUPPLEMENTARY MATERIALS FIGURE AND TABLES



## Abbreviations

BAT: brown adipose tissue
CGA: chromogranin A
CD56: cluster of differentiation 56
CSA: cross-sectional area
CEA: carcinoembryonic antigen
CLEIA: chemiluminescent enzyme immunoassay
D-NEC: neuroendocrine carcinoma of the duodenum
DMEM: Dulbecco’s modified eagle medium
ELISA: enzyme-linked immunosorbent assay
GEP: gastroenteropancreatic
HGF: hepatocyte growth factor
IHC: immunohistochemistry
IL: interleukin
NET: neuroendocrine tumor
NGS: next generation sequence
NSE: neuron specific enolase
OI: orthotopic implantation
PD-NEC: poorly differentiated neuroendocrine carcinoma
PBS: phosphate buffered saline
RB: retinoblastoma
SSTR: somatostatin receptor
STR: short tandem repeat
s.c.: subcutaneous
i.p.: intraperitoneal
SYN: synaptophysin
TV: tumor volume
VEGF: vascular endothelial growth factor
WAT: white adipose tissue
