# Supplementary material for: Development and characterization of a cancer cachexia model employing a rare human duodenal neuroendocrine carcinoma-originating cell line
Source: Oncotarget. 2019 Mar 29;10(25):2435–50. doi: 10.18632/oncotarget.26764 (PMC6497432; doi:10.18632/oncotarget.26764)
Supplement: Supplementary file 1 [file oncotarget-10-2435-s001.pdf]

Development and characterization of a cancer cachexia model employing a rare human duodenal neuroendocrine carcinoma-originating cell line

SUPPLEMENTARY MATERIALS

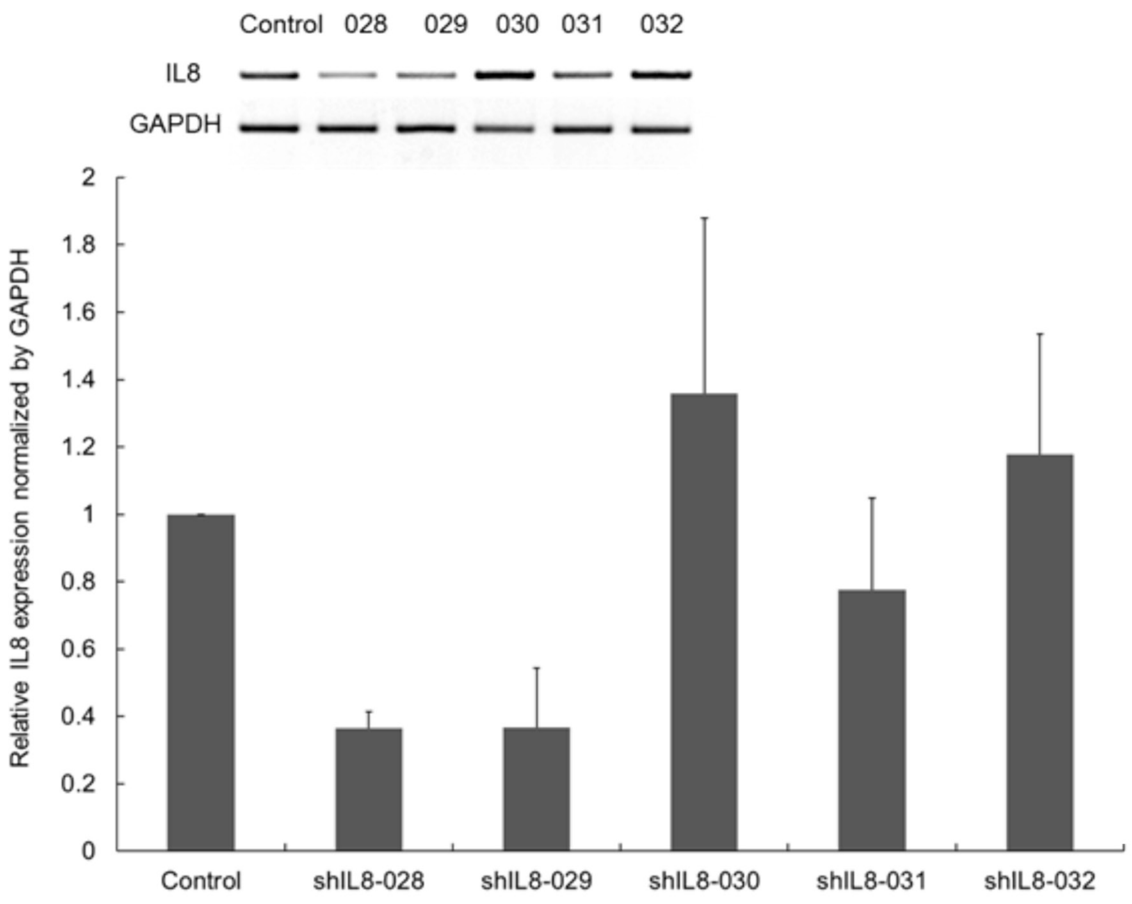

Supplementary Figure 1: Generation of IL-8-specific shRNA-expressing AkuNEC cells. The lentiviral vector-expressed shRNA for silencing human IL-8 (NM 000584) were designed and synthesized by Sigma-Aldrich. Five individual clones from shRNA targets for IL-8 inserted into a lentivirus packaging plasmid (MISSION™ lentiviral transduction particles, SHCLNV, Sigma-Aldrich) were used to infect the AkuNEC cell line. The target sequences of five individual shRNA (shIL8-028, shIL8-029, shIL8-030, shIL8-031, and shIL8-032) for IL-8 are shown in Supplementary Table 1. After 24 hours post-infection, the shRNA lentiviral particle-containing medium was removed and replaced with fresh medium containing puromycin (1 µg/mL) for selection of transduced cells. To analyze shRNA efficacy, cells were harvested and the mRNA level of IL-8 was detected by real-time PCR. Glyceraldehyde-3-phosphate dehydrogenase (GAPDH) mRNA was used as an internal control. The AkuNEC cells used in this study that expressed IL-8 target shRNA (AkuNECshIL8) were chosen for the clone that showed the highest RNAi potency for IL-8 (shIL8-028). AkuNEC cells expressing non-target shRNA, which were infected by MISSION™ pLKO.1-puro non-target shRNA control transduction particles (SHC016V), were used as control cells (AkuNECshcontrol).

Supplementary Table 1: Target sequence of IL-8 for shRNA

shRNA	shRNA insert sequence
shIL8-028	ccggCCGAACTTTAATTTCAGGAATctcgagATTCCTGAAATTAAAGTTCGGtttttg
shIL8-029	ccggGCCAACACAGAAATTATTGTActcgagTACAATAATTTCTGTGTTGGCtttttg
shIL8-030	ccggCAAGGAGTGCTAAAGAACTTActcgagTAAGTTCTTTAGCACTCCTTGtttttg
shIL8-031	ccggGCTCTGTGTGAAGGTGCAGTTctcgagAACTGCACCTTCACACAGAGCtttttg
shIL8-032	ccggCTGCGCCAACACAGAAATTATctcgagATAATTTCTGTGTTGGCGCAGtttttg
shcontrol	ccggGCGCGATAGCGCTAATAATTTctcgagAAATTATTAGCGCTATCGCGCttttt

The match sequences of shRNA are indicated in uppercase letters, non-match sequences are indicated in lowercase letters and the loop region of shRNA are indicated in lowercase letters with underline.

Supplementary Table 2: Target genes and sequence of primers used for real-time PCR (RT-PCR)

Genes	Forward (5′ → 3′)	Reverse (5′ → 3′)
IL-8	AAGCTGGCCGTGGCTCTCTT	TGGTGGCGCAGTGTGGTCCA
GAPDH	ACGACCAAATCCGTTGACTC	GCTCTCTGCTCCTCCTGTTC

The level of gene expression was evaluated by measuring target mRNAs quantitatively using reverse transcription and real-time PCR. Glyceraldehyde-3-phosphate dehydrogenase (GAPDH) mRNA was also measured as an intrinsic control. The total RNA was extracted using an RNeasy Plus Mini Kit (Qiagen, Valencia, CA), and the yield was determined by measuring the optical density at a wavelength of 260 nm on a UV spectrometer. Complementary DNA was synthesized using the PrimeScript™ RT reagent Kit (Takara, Shiga, Japan) using 20 µg/mL of extracted total RNA. Real-time PCR was performed using a Thermal Cycler Dice Real Time System II (Takara) with the SYBR Premix EX Taq™ II (Takara) reagent according to the manufacturer's protocol.
